# Supplementary material for: Composition of cetacean communities worldwide shapes their contribution to ocean nutrient cycling
Source: Nat Commun. 2023 Sep 19;14:5823. doi: 10.1038/s41467-023-41532-y (PMC10509247; doi:10.1038/s41467-023-41532-y)
Supplement: Supplementary file 2 — Description of Additional Supplementary Files [file 41467_2023_41532_MOESM2_ESM.pdf]

<u>Description of Additional Supplementary Files Document</u>

Supplementary Data 1: Summary statistics of outputs of the bioenergetic model analysis, at several levels.

Supplementary Data 2: Statistical test results for comparison between model outputs at several levels.

Supplementary Data 3: Summary statistics of model parameters and model intermediate parameters as a result of Monte Carlo simulations and bootstrapping.

Supplementary Data 4: References used for the setting of model parameters and input data for abundance data and diet description.
