# Supplementary material for: Composition of cetacean communities worldwide shapes their contribution to ocean nutrient cycling
Source: Nat Commun. 2023 Sep 19;14:5823. doi: 10.1038/s41467-023-41532-y (PMC10509247; doi:10.1038/s41467-023-41532-y)
Supplement: Supplementary file 7 — Reporting Summary [file 41467_2023_41532_MOESM7_ESM.pdf]

Corresponding author(s): Jérôme Spitz

Last updated by author(s): Aug 21, 2023

# Reporting Summary

Nature Portfolio wishes to improve the reproducibility of the work that we publish. This form provides structure for consistency and transparency in reporting. For further information on Nature Portfolio policies, see our Editorial Policies and the Editorial Policy Checklist.

## Statistics

For all statistical analyses, confirm that the following items are present in the figure legend, table legend, main text, or Methods section.

| n/a | Confirmed | |
|---|---|---|
| ☐ | ☒ | The exact sample size (*n*) for each experimental group/condition, given as a discrete number and unit of measurement |
| ☐ | ☒ | A statement on whether measurements were taken from distinct samples or whether the same sample was measured repeatedly |
| ☐ | ☒ | The statistical test(s) used AND whether they are one- or two-sided<br>*Only common tests should be described solely by name; describe more complex techniques in the Methods section.* |
| ☐ | ☒ | A description of all covariates tested |
| ☐ | ☒ | A description of any assumptions or corrections, such as tests of normality and adjustment for multiple comparisons |
| ☐ | ☒ | A full description of the statistical parameters including central tendency (e.g. means) or other basic estimates (e.g. regression coefficient) AND variation (e.g. standard deviation) or associated estimates of uncertainty (e.g. confidence intervals) |
| ☒ | ☐ | For null hypothesis testing, the test statistic (e.g. *F*, *t*, *r*) with confidence intervals, effect sizes, degrees of freedom and *P* value noted<br>*Give P values as exact values whenever suitable.* |
| ☒ | ☐ | For Bayesian analysis, information on the choice of priors and Markov chain Monte Carlo settings |
| ☒ | ☐ | For hierarchical and complex designs, identification of the appropriate level for tests and full reporting of outcomes |
| ☒ | ☐ | Estimates of effect sizes (e.g. Cohen's *d*, Pearson's *r*), indicating how they were calculated |

*Our web collection on statistics for biologists contains articles on many of the points above.*

## Software and code

Policy information about availability of computer code

| | |
|---|---|
| Data collection | No data was collected for this study. |
| Data analysis | R Core Team (2021) was used for all data analysis described in the manuscript. It is cited in the Methods section. |

For manuscripts utilizing custom algorithms or software that are central to the research but not yet described in published literature, software must be made available to editors and reviewers. We strongly encourage code deposition in a community repository (e.g. GitHub). See the Nature Portfolio guidelines for submitting code & software for further information.

## Data

Policy information about availability of data

All manuscripts must include a data availability statement. This statement should provide the following information, where applicable:

- Accession codes, unique identifiers, or web links for publicly available datasets
- A description of any restrictions on data availability
- For clinical datasets or third party data, please ensure that the statement adheres to our policy

No original data were used nor generated for this analysis which mobilised data from the literature for the setting of a majority of parameters in our bioenergetic model. All sources are provided in the manuscript and in Supplementary Data 4, the latter including also means and coefficient of variations used to define population abundances and diet description for each species.. The external datasets of prey composition are available at https://doi.pangaea.de/10.1594/PANGAEA.937345 and https://doi.pangaea.de/10.1594/PANGAEA.940861 (see Methods). Oceanographic data was downloaded at https://

oceancolor.gsfc.nasa.gov/l3/order/ in March 2022 and download procedure is indicated in a ReadMe file in the corresponding data folder, on the Github repository attached to the study (https://github.com/Lola-san/Cetacean.excretion.global.git).

## Human research participants

Policy information about studies involving human research participants and Sex and Gender in Research.

| | |
|---|---|
| Reporting on sex and gender | Not applicable |
| Population characteristics | Not applicable |
| Recruitment | Not applicable |
| Ethics oversight | Not applicable |

Note that full information on the approval of the study protocol must also be provided in the manuscript.

# Field-specific reporting

Please select the one below that is the best fit for your research. If you are not sure, read the appropriate sections before making your selection.

☐ Life sciences ☐ Behavioural & social sciences ☒ Ecological, evolutionary & environmental sciences

For a reference copy of the document with all sections, see nature.com/documents/nr-reporting-summary-flat.pdf

# Ecological, evolutionary & environmental sciences study design

All studies must disclose on these points even when the disclosure is negative.

**Study description**

This is a modelling study estimating quantities of nutrients released in surface water by whole cetacean communities (i.e. in their waste products urine and feces) in 14 areas situated in the Northern emisphere, and in subtropical and tropical areas of the world of the Atlantic, Indian and Pacific ocean. The study is based on the use of a bio-energetic model estimating the daily release of nutrients by an individual cetacean given its daily energetic requirements, its food consumption, its diet and the nutrient composition of its food items. Estimates were then raised to whole populations in each area of interest.The 8 parameters of the model (individual body mass, metabolic index, assimilation efficiency, nutrient release rate, population abundance, number of days of presence in the area, mean energy content of the diet, mean nutrient content of the diet) were associated with either Monte-Carlo simulations or bootstrap drawings to account for uncertainty in their values based on available data from the litterature. 10,000 simulations were used for both Monte Carlo simulations and the bootstrap procedure, to simulate variability in all model parameters, i.e. simulate possible states of each species' population as described with our parameters. We fully described the setting of parameters in the Methods section.
Level of estimated amounts of nutrient released were compared between areas, and between habitats within each areas (i.e. oceanic versus neritic waters). These model outputs were also compared to indicators of productivity in each area (sea surface temperature and surface chlorophyll concentration). Considering three cetacean ecological groups (baleen whales, deep divers and small delphinids), we compared their levels of nutrient release, at the individual (using Principal Component Analysis) and community level (using statistical tests described in the Methods).

**Research sample**

Most of the data used in the analysis (i.e. for the setting of the model parameters) were collected from the litterature. This include data on cetacean population abundance, species body mass, diet, ecology and physiology, and prey energy and nutrient content. For population abundance estimates, we restricted our analysis to full cetacean community levels. We used only cetacean abundance estimates inferred from large scale multi-species surveys. We used only estimates from large scale distance sampling surveys conducted either from boat or plane, and analysed using a similar design-based procedure. This criteria restricted the number of areas included in the study. Species body mass was inferred from available data in the litterature, either resulting of direct measurements or of estimation from maximum body length. For species diet, we searched for all available data but used quantitative data from stomach content analysis whenever available. As we used a description of diet using ecological group of preys and not directly prey species, we could infer a broad diet using these groups when no quantitative data was available, based on the qualitative data available (e.g. isotope studies or direct observations). For poorly known species for which even qualitative data was not found, we used the diet of a species identified to have a similar trophic ecology. As we included a wide range of essential nutrients in our study, we were restricted in the data available from the litterature on the energy and nutrient content of prey worldwide, that is usually based on the analysis of few nutrients. We therefore used only two datasets of elemental concentrations that include 154 species of prey sampled from the Bay of Biscay, Northeast Atlantic. We then used a kernel-based bootstrap procedure to infer about the elemental composition of prey groups,to account for uncertainty and variability of the composition of prey items belonging to each of them. Assimilation efficiency was set based on available data from the litterature (based on experimental studies with captive pinnipeds, mostly) and common practice in modelling studies. For nutrient release rates, we expanded our research to all mammals, as for micronutrients very little information was available. We set these parameters based on the general knowledge of nutrient balance physiology in mammals we found in the litterature. All references used for the setting of model parameters (i.e. distribution, base values and variation parameter) and describing the samples used in the analysis are included in supplementary Data 4, and the processes briefly described here are described in the Methods section of the manuscript. The statistics associated to each parameter, after Monte-Carlo and bootstrap procedures, are given for each species in Supplementary Data 3.

| | |
|---|---|
| | For the comparison between levels of nutrient release and indicators of ecosystem productivity, we used spatial estimates from satellite data available at https://oceancolor.gsfc.nasa.gov. We used spatialised average values for year 2021, and computed basic statistic over each area (mean and confidence intervals). |
| Sampling strategy | A sample size of 10,000 was chosen for Monte-Carlo simulations, or 10,000 bootstrap drawings, as an optimal value to get a sufficient number of simulations to provide reliable and reproducible results but requiring a manageable model running time. The analysis was run several times with a smaller sample size (1,000) in the development of the analysis before being set to 10,000, but results were most stable with this sampling size given that we use common Distributions (Normal, truncated Normal, logNormal, Uniform, ...). This sample size is greater than the one used in most modelling studies in ecology. |
| Data collection | As mentioned above, data for the setting of parameters was collected from literature research conducted by Lola Gilbert, using mostly Google scholar as well as direct exchange with researchers. Data on prey composition was already available, and DOI are provided in the manuscript (Methods and Data Availability sections) and in the present Reporting Summary (Data section). |
| Timing and spatial scale | Literature research was conducted between October 2020 and July 2021. Spatial scale was set based on the large scale cetacean abundance surveys selected. It thus varied between areas from 110,600 km2 to 2,790,700 km2. To account for differences in the surfaces of each area we used spatial densities of amounts of nutrient released per year by cetacean for any of the comparative approaches conducted. Surveys were conducted between year 2009 and 2018. |
| Data exclusions | Data was excluded only when using linear models to test correlations between levels of nutrient release and productivity in each area. French Guyana is close to the mouth of the Amazon river which largely influence the turbidity of its waters. We think estimated surface chlorophyll concentrations could be influence by these processes (they are largely over values of nearby areas and suprisingly high for a tropical area). We therefore excluded French Guyana in the linear model comparing levels of nutrient released with mean surface chlorophyll concentration. This is mentioned in the corresponding figure legend (Fig 2). |
| Reproducibility | We used good coding practices offered by R software (e.g. the use of R projects) and R packages (e.g. "targets") to be confident about the reproducibility of our results. All steps of the analysis were separated in different scripts and all were associated to R functions. We used Git and Github to track every step of modification of the code of the analysis, and all is made available on the Github repository associated to the manuscript (https://github.com/Lola-san/Cetacean.excretion.global.git). The analysis was run several times with similar results. |
| Randomization | This is relevant for the allocation of prey species to prey groups (e.g. small energy-rich schooling fish, large demersal fish, bottom cephalopods, etc). This was based on the ecology or the composition of the prey species, either from the litterature or personal knowledge of one the co-author, namely Jérôme Spitz. |
| Blinding | *Describe the extent of blinding used during data acquisition and analysis. If blinding was not possible, describe why OR explain why blinding was not relevant to your study.* |

Did the study involve field work? ☐ Yes ☒ No

# Reporting for specific materials, systems and methods

We require information from authors about some types of materials, experimental systems and methods used in many studies. Here, indicate whether each material, system or method listed is relevant to your study. If you are not sure if a list item applies to your research, read the appropriate section before selecting a response.

## Materials & experimental systems

| n/a | Involved in the study |
|---|---|
| ☒ | ☐ Antibodies |
| ☒ | ☐ Eukaryotic cell lines |
| ☒ | ☐ Palaeontology and archaeology |
| ☒ | ☐ Animals and other organisms |
| ☒ | ☐ Clinical data |
| ☒ | ☐ Dual use research of concern |

## Methods

| n/a | Involved in the study |
|---|---|
| ☒ | ☐ ChIP-seq |
| ☒ | ☐ Flow cytometry |
| ☒ | ☐ MRI-based neuroimaging |
